# Cooperation between Angiogenesis, Vasculogenesis, Chemotaxis, and Coagulation in Breast Cancer Metastases Development: Pathophysiological Point of View

**DOI:** 10.3390/biomedicines10020300

**Published:** 2022-01-27

**Authors:** Elżbieta Zarychta, Barbara Ruszkowska-Ciastek

**Affiliations:** Department of Pathophysiology, Faculty of Pharmacy, Nicolaus Copernicus University, Collegium Medicum in Bydgoszcz, 85-094 Bydgoszcz, Poland

**Keywords:** breast cancer, metastasis, angiogenesis, vasculogenesis, chemotaxis, coagulation, VEGF-A, CXCL12/CXCR4, tissue factor, endothelial progenitor cells

## Abstract

**Simple Summary:**

Breast cancer is one of the main causes of morbidity and mortality in women. Early breast cancer has a relatively good prognosis, in contrast to metastatic disease with rather poor outcomes. Metastasis formation in distant organs is a complex process requiring cooperation of numerous cells, growth factors, cytokines, and chemokines. Tumor growth, invasion, and finally systemic spread are driven by processes of angiogenesis, vasculogenesis, chemotaxis, and coagulation. This review summarizes their role in development of distant metastases in breast cancer, as well as explains the essential processes occurring throughout these actions.

**Abstract:**

With almost 2.3 million new cases and 685 thousand fatal events in 2020 alone, breast cancer remains one of the main causes of morbidity and mortality in women worldwide. Despite the increasing prevalence of the disease in recent years, the number of deaths has dropped—this is mostly the result of better diagnostic and therapeutic opportunities, allowing to recognize and treat breast cancer earlier and more efficiently. However, metastatic disease still remains a therapeutic challenge. As mechanisms of tumor spread are being explored, new drugs can be implemented in clinical practice, improving the outcomes in patients with advanced disease. Formation of metastases is a complex process, which involves activation of angiogenesis, vasculogenesis, chemotaxis, and coagulation. The actions, which occur during metastatic spread are interrelated and complementary. This review summarizes their importance and mutual connections in formation of secondary tumors in breast cancer.

## 1. Introduction

### 1.1. General Information Related to Breast Cancer

**Figure 1:**
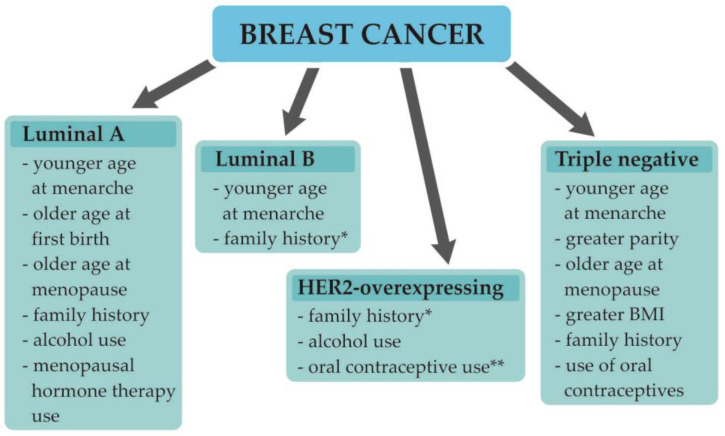
Breast cancer molecular subtypes and their risk factors [4]. Abbreviations: BMI, body mass index; * there is insufficient or inconsistent evidence of an association between the luminal B breast cancer subtype and greater parity, older age at first birth, older age at menopause, greater BMI, alcohol use, use of oral contraceptives, or menopausal hormone therapy use; ** there is insufficient or inconsistent evidence of an association between HER2-overexpressing breast cancer and younger age at menarche, greater parity, older age at first birth, breastfeeding, older age at menopause, greater BMI, or menopausal hormone therapy use.

Breast cancer (BC) is in first place in terms of the most commonly diagnosed malignancies in women worldwide, with almost 2.3 million new cases and 685 thousand deaths in 2020 alone [1]. As the incidence of BC in recent years has increased, the prognosis for patients has improved as a result of progress in early diagnosis and treatment approaches, leading to a drop in mortality from BC in North America and the European Union [2,3]. The rise in the number of new cases in developing countries is attributed mostly to lifestyle changes, diet, environment alterations, older gestational ages, alcohol consumption, and the use of menopausal hormone therapy [2]. The established BC risk factors include being female, early age at menarche and older age at menopause, nulliparity, no breastfeeding, higher body mass index, family history of BC, alcohol use, and use of contraceptives or menopausal hormone therapy. However, as BC is a complex disease, for particular BC subtypes, the presence of some of these factors may be not associated with an increased risk of the disease, or they could be even considered protective (Figure 1) [4].

### 1.2. Understanding Breast Cancer Heterogeneity

BC is a complex disease with miscellaneous morphologic and biological features and, hence, various clinical courses and prognoses [5]. The traditional classification of BC is based on tumor stage and grade. The TNM staging system by the American Joint Committee on Cancer encompasses both clinical and pathologic data on tumor size (T), the status of regional lymph nodes (N), and distant metastases (M); subsequently, based on these data, the disease is established as being in one of five stages (0–IV) [5]. Tumor grade brings information on cell differentiation—it includes evaluation of the particular features of the cell, which include tubule formation, size and shape of the nucleus in tumor cells, and mitotic rate [5]. With more than 20 different histologic types; BC can be classified based on clinicopathologic features such as cell type of the tumor, extracellular secretion, architectural features, or immunohistochemical profile. The most common type is invasive ductal carcinoma not otherwise specified (IDC-NOS), which is found in 70–80% of cases. It encompasses adenocarcinomas that cannot be classified as one of the special types [5,6]. The most common among special types of BC is invasive lobular carcinoma, accounting for about 10% of cases. Other less common special types include: tubular, cribiform, mucinous, papillary, apocrine, medullary, metaplastic, or mixed type carcinomas [5,6]. In recent decades, there has been meaningful development in the understanding of BC biology, which has led to the conclusion that the previous classifications do not display the heterogeneity of the disease—both response to therapy and prognosis are defined by the biological features of a tumor [5,7]. Routine immunohistochemical analysis of estrogen receptor (ER), progesterone receptor (PR), human epidermal growth factor receptor 2 (HER2), and Ki-67 expression (proliferation index) is currently used to determine the four basic molecular subtypes of BC: Luminal A-like, luminal B-like, HER2-overexpresing, and triple-negative BCs. This classification (approved by the St Gallen Consensus Conference) facilitates choosing the right therapeutic approach and allows to determine a patient’s future outcome [5,8]. Apart from the molecular subtype of BC, the TNM stage and the patient’s preferences, as well as many other factors, play a role in choosing the most personalized therapy. The clinico-prognostic characteristics of particular subtypes are shown in Table 1. The first-line therapy for the early BC combines surgery and postoperative radiation. Main surgical approaches include mastectomy or an excision followed by radiation. Breast cancer surgery may also include dissection of axillary lymph nodes if there are indications for the procedure. Furthermore a pre- (neoadjuvant) or postoperative (adjuvant) systemic therapy may be administered, which includes chemotherapy, endocrine therapy, or trastuzumab-based therapy. In metastatic disease, the major objectives are prolongation and maintaining quality of life. The systemic therapy encompasses chemotherapy, endocrine therapy, trastuzumab-based therapy, or other targeted therapies such as CDK4/6 inhibitor or PARP inhibitor [9]. In some patients, breast surgery may be implemented as it could improve local progression free survival; however, it may worsen distant progression free survival. There is no evidence that surgical treatment of the primary tumor improves overall survival in metastatic disease [10].

### 1.3. Breast Cancer Development, Progression, and Metastasis Formation

BC development is a complex and intricate process that depends on genetic and epigenetic alterations, as well as on the tumor microenvironment. Cancer cells are surrounded by a modified stroma, which creates the tumor microenvironment (TME). It comprises miscellaneous stromal cells, encompassing fibroblasts, immune cells, inflammatory cells, endothelial cells (ECs), pericytes, adipocytes, and bone marrow-derived cells [2,12,13]. Both tumor and stromal cells interact mutually through secreted proteins, cytokines, chemokines, and growth factors, which lead to BC growth, progression, and metastasis formation [14,15,16].

Early diagnosis and targeted therapy are the main factors that, in recent decades, have allowed for improvement in the overall survival of BC patients. As non-metastatic BC is considered to be generally curable with a five-year overall survival greater than 80%, in metastatic disease, only palliative treatment is applicable, with a reduction in the five-year overall survival rate of only 25% [15,16,17].

Metastasis formation is a multi-step and complex process, which implies cooperation between angiogenesis, vasculogenesis, chemotaxis, and coagulation. During this action, cancer cells acquire proangiogenic phenotype and are able to escape from their initial location, leading to remodeling the extracellular matrix (ECM), intravasate into the blood, survive in the circulation, and extravasate from blood vessels to colonize new organs. In recent years, it has been speculated that BC cells spread systematically during the early disease stage, regardless of the primary tumor size [18]. During cancer development, numerous cell types continuously liberate signaling molecules into the microenvironment, leading to the formation of a sophisticated web of communication that attracts new cell types to remodel the tumor microenvironment [19,20].

### 1.4. The Aim of This Review

In this review, we aimed to discuss previous and current studies investigating the angiogenesis, vasculogenesis, chemotaxis, and coagulation processes and their role in the formation of distant metastases in bones, lungs, the liver, and the brain in BC patients. Furthermore, we wanted to emphasize their mutual relationships in cancer development and progression.

## 2. Angiogenesis

### 2.1. The Role of Angiogenesis in Tumor Invasion

Angiogenesis is the process of new blood vessels development from preexisting vasculature. This process occurs during physiological processes such as wound healing or endometrial growth during the menstrual cycle, and can also be part of a disease; for instance, in cancer transformation, when tumor diameter exceeds 1–2 mm^3^, it requires its own blood supply. Angiogenesis also plays an important role in cancer dissemination and the formation of metastases [21,22]. The initiation of angiogenesis—the so-called angiogenic switch—is the point in tumor development where proangiogenic factors surpass antiangiogenic factors and a progressive tumor growth is initiated. The angiogenic switch is set off either by genetic mutations of tumor cells, resulting in increased proliferation and hypoxia or the expression of proangiogenic factors, or by tumor-associated inflammation and the recruitment of immune cells [23,24].

### 2.2. The Role of Angiogenesis in BC

Hypoxia plays a major role in the stimulation of angiogenesis in BC. Endothelial cells (ECs) start expressing hypoxia-inducible factor-1 (HIF-1) as a result of low intratumoral oxygen levels. Furthermore, overexpression of HER2 and expression of estrogen receptor on BC cells is associated with increased HIF-1 levels. High HIF-1 levels have been also found in triple-negative breast cancer, which is probably a result of loss of p53, *PTEN* mutations, and EGFR overexpression [22]. HIF-1 upregulation leads to the transcription of genes involved in adaptation to the hypoxic BC environment [21,22,25]. Hypoxia, via HIF-1, stimulates cancer cells to secrete large amounts of vascular endothelial growth factor A (VEGF-A), which binds to its receptor on the surface of ECs. This leads to the activation, growth, and migration of ECs toward the tumor [26]. The migrating cells start to proliferate and subsequently form the lumen of the new vessel, which supplies cancer cells with oxygen and nutrients [21]. This process is supported by various cytokines, enzymes, and growth factors (Table 2). Furthermore, as VEGF-A has the ability to mobilize endothelial progenitor cells (EPCs) from bone marrow, it facilitates formation of new blood vessels de novo via vasculogenesis [27].

### 2.3. Interactions of VEGF with Tumor and Tumor Microenviroment

Elevated levels of VEGF-A in BC are linked to aggressive tumor behavior and poor prognosis [26,37]. Tumors overexpressing HER2 also over-release VEGF-A. The poor prognosis of patients with HER2-overexpressing tumors is linked to excessive angiogenesis [38,39]. Close cooperation between proangiogenic factors (including VEGF-A), their receptors, and the components of the ECM matrix is demanded in angiogenesis in BC [22]. There are numerous factors that stimulate angiogenesis. These include estradiol and progesterone. Estradiol upregulates VEGF-A expression by vascular epithelium and supports EC proliferation and migration [40]. Cancer-associated fibroblasts (CAFs) are one of the main components of the TME and boost cancer growth and metastasis formation. They secrete tumor-promoting mediators, causing remodeling of the ECM, thus supporting the invasion of cancer [41]. Another component of the TME in BC is tumor-associated macrophages (TAMs), which promote angiogenesis and tumor progression via releasing a proangiogenic factor, CCL18 [28].

### 2.4. Contribution of VEGF to the Metastatic Spread of BC

VEGF-A binds to its receptors on ECs—either to VEGFR1 or VEGFR2. VEGFR2 is the major receptor for VEGF-A, and its activation leads to the proliferation of ECs, invasion, migration, and survival (via the ERK and PI3K/Akt pathways) [24]. VEGFR1 is found not only on ECs, but also on several other cell types, including monocytes or macrophages [42]. In BC it activates MAPK/ERK and PI3K/Akt pathways, resulting in tumor growth and induction of epithelial-to-mesenchymal transition (EMT) [43]. PI3K/Akt may trigger nuclear factor-kappa B (NF-κB) to activate migration pathway. Furthermore, AKT pathway stimulates permeability factors and inactivates proapoptotic factors [44]. Presence of receptors for VEGF on BC cells is linked to cancer cells proliferation, survival, migration, adhesion, and invasion as a result of autocrine signaling pathway activation [45]. Interestingly, in small metastatic lesions the extensive activation of proangiogenic factors—the angiogenic switch—is necessary for the progression of small avascular micrometastases to macrometastases and thus escaping of cancer cells from dormancy [46]. The soluble form of VEGFR1 (sVEGFR1), which is a product of the short mRNA transcript of the VEGFR1 gene, impairs angiogenesis—it binds to VEGF-A, thus preventing it from binding to VEGFR1 or VEGFR2 on ECs and inhibiting signal transduction [40,42].

### 2.5. Structure of Tumoral Vessels

Newly formed blood vessels may serve as a gate for cancer cells to enter the systemic circulation, as they are fairly distinct from normal blood vessels [25]. Partially, it is caused by the structure of tumor-associated capillaries—they are abnormally formed, being circuitous or twisted, instead of bifurcated hierarchical figures found in regular capillaries. The ECs in these vessels are morphologically immature and irregular, with cytoplasmic extensions to the lumen. Tumor blood vessels are fenestrated, with endothelial gaps, thus making them highly permeable. Moreover, most of these capillaries lack a pericyte layer or their endothelial cells are loosely bound to the pericyte layer, different than in normal vessels [21,47]. All of this favors cancer cell extravasation, dissemination, and metastasis formation.

## 3. Vasculogenesis

### 3.1. Alternative Method of Tumor Vessel Formation

In embryos, the vasculature is formed in a process of vasculogenesis. This requires the involvement of endothelial progenitor cells (EPCs) derived from bone marrow, which differentiate into ECs to form a primary capillary network [24,48]. EPCs can also support physiological and pathological vessel formation in adults [48]. EPCs constitute 0.002% of the total mononuclear cells in the peripheral blood of healthy individuals [49].

Neovascularization involves the development of all types of new blood vessels during the entire postnatal lifetime [50]. The formation of new vessels within a tumor comprises angiogenesis and vasculogenesis—the process of the de novo formation of blood vessels involving EPCs [40,50,51]. EPCs are a subtype of stem cells that are involved in some processes of tissue repair such as myocardial ischemia and infarction, limb ischemia, and wound healing [49]. Furthermore, low oxygen levels in sites such as fetal liver, umbilical cord, and tumor tissues can trigger EPCs mobilization [49]. Circulating EPCs can be generally divided into two subpopulations: early and late. Early EPCs promote new vessels formation by paracrine secretion of a number of growth factors and cytokines such as VEGF, CXCL12, insulin-like growth factor 1, but late ones are able to mature into functional endothelial cells [52].

### 3.2. Role of EPCs in Breast Cancer

EPCs are a subtype of stem cells that have the ability to self-renew, proliferate, mediate neovascularization, and rebuild endothelial tissue, and are suspected to support early stages of BC development [53]. The number of EPCs is increased in the blood of BC patients [50]. Patients with advanced BC are characterized by an increased number of EPCs compared to those with an early-stage disease [54]. The amount of EPCs is suspected to be a prognostic factor for BC patients; however, the results of such studies remain contradictory [55,56]. It was found that patients with good response to systemic therapy had greater drop of circulating EPCs [57]. Some cytotoxic drugs could mobilize EPCs from the bone marrow to the tumor site, enhancing formation of new blood vessels [58]. This could also happen during anti-angiogenic therapy, which might be a possible explanation of resistance to anti-angiogenic therapy in some cases [59]. There are various sources of EPCs, which include hematopoietic stem cells, myeloid cells, circulating mature ECs, and other circulating progenitor cells [24]. In BC, EPCs are mobilized from the bone marrow and move to the tumor site, stimulated by chemotaxis [40]. The main surface markers of EPCs are CD34, CD309 (VEGFR2/KDR), and CD133 [49], but they can also express VE-cadherin, CXCR4, CD31, CD105, CD144, CD106, and CD117 [60].

### 3.3. Role of Hypoxia and Inflammation in EPCs Mobilization and Homing

Hypoxia is the main driver of EPCs homing at the tumor site—this induces, via HIF-1α expression, numerous growth factors, cytokines, and chemokines, including VEGF-A, CXCL12, and CXCR4 [40]. These molecules, along with hypoxia and chronic inflammation, engage EPCs [40].

Other factors such as chemokines CCL2 or CCL5 and adiponectin also contribute to EPC mobilization [24]. Furthermore, VEGF-A shifts EPCs to the tumor site by binding the VEGFR1 and VEGFR2 found on EPCs [60]. ECs, together with immune cells, release paracrine factors, favoring EPCs at the tumor site. EPCs are attracted by the CXCL12 gradient into a tumor’s hypoxic microenvironment [49]. Moreover, CXCL12 increases vascular permeability and reduces endothelial tight junction proteins, facilitating their recruitment from bone marrow [19].

Thus far, both CXCL12/CXCR4 and VEGF/VEGFR are the dominant pathways of EPC mobilization from bone marrow in cancer development [60]. Chronic inflammation at the tumor site also attracts EPCs and pericyte progenitors from bone marrow to the inflammation site [49].

### 3.4. Interactions of EPCs with Tumor and Tumor Microenviroment

Recruitment of EPCs from circulation to the tumor bed is an essential step in the formation of new vessels in the process of neoplastic vasculogenesis. Circulating EPCs follow a CXCL12 gradient towards the tumor bed and then integrate into the nascent vessels [52]. After homing, EPCs secrete proangiogenic factors and inflammatory cytokines, leading to exacerbation of angiogenesis and inflammation processes [40]. EPCs lead to tumor progression through increasing production of VEGF-A, CXCL12, or CXCR4 [49]. On the other hand, VEGF-A enhances proliferation of EPCs via activation of PI3K/Akt/eNOS pathway, leading to an increase of NO level, subsequently resulting migration and proliferation of EPCs and tubule formation [49]. Undoubtedly, all those reactions intensify the vicious cycle between vasculogenesis, angiogenesis, and chemotaxis, which lead to uncontrolled cancer cells proliferation and enhanced tumor mass. Furthermore, EPCs might be activated on particular sites before arrival of tumor cells, contributing to formation of pre-metastatic niche [49].

## 4. Chemotaxis

### 4.1. The Role of Chemotaxis in Tumor Invasion

Chemotaxis is one of the key actions that occurs during cancer development, progression, and metastasis formation. Chemokines are signaling proteins that activate chemotaxis by controlling cell movement and stimulating proliferation [8,20]. They are involved in homeostasis and inflammation processes. In BC, chemokines and their receptors control tumor progression and metastasis formation [61] by promoting cell motility, invasion, interactions with the ECM, and cell survival [61].

### 4.2. The Role of Chemotaxis in BC

The concentration of CXCL12 is suggested to be a prognostic factor for BC patients; however, the results of such studies remain divergent [62,63]. Its expression is linked to the malignant behavior and poor prognosis of tumors [61,62,64,65]. On the other hand, overexpression of the CXCL12 is associated with a better prognosis and longer survival rates. Since, higher expression of CXCL12 was linked with a positive ER status, a negative HER2 status, and a small tumor size, this confirms the role of CXCL12 as a favorable prognostic factor [52,66]. The reason for the opposite effect of high CXCL12 expression in BC is unclear and still poorly understood. It has been suggested that this difference may be due to the clinical and biological heterogeneity of BC in respect to other cancer types. BC is rarely fatal unless it is metastatic, while esophagus, stomach, lung, and pancreas cancers are often terminal due to local tumor invasion. CXCL12 is able to promote local invasion of neoplastic cells, while loss of CXCL12 expression in primary tumor site supports the migration of cancer cells to organs exhibiting high amount of CXCL12. Therefore, the unfavorable effect of SDF-1α in BC may be a suppression of SDF-1α expression in the tumor environment to promote metastasis, while in other cancers, high SDF-1α expression might be associated with a poor prognosis since an increase in SDF-1α promotes local invasion and consequently leads to an increase in mortality. Nevertheless, further research is needed to elucidate the role of SDF-1α in breast cancer progression [66].

### 4.3. Interactions of CXCL12 with Tumor and Tumor Microenviroment

The CXCL12/CXCR4 axis is of major significance in BC development. In contributes to tumor growth, metastasis, chemotaxis, and angiogenesis [67,68]. CXCL12 is released from cancer and cancer-adjacent stromal cells, mainly from fibroblasts [61]. It exerts its actions by binding to receptor CXCR4, which can be found on distinct types of cells, including ECs and EPCs [20]. Normal breast tissue does not express CXCR4, but it can be found on BC cells, as well as on other cancerous cells, such as kidney, lung, brain, or prostate cancers. Moreover, expression of CXCR4 can be induced by a potent proangiogenic factor—VEGF [30].

CXCL12 activates fibroblasts via the CXCR4 receptor and stimulates their conversion into CAFs, which are one of the main drivers of infiltration, growth, and angiogenesis in the TME. They support the invasive growth of tumors, as opposed to normal fibroblasts, which block tumor formation [69,70]. CAFs trigger ECs to release CXCL12 to attract CXCR4-expressing cells, mainly EPCs, thus promoting angiogenesis. Furthermore, they increase vascular permeability by decreasing the expression of endothelial tight junctions and adherence junction molecules, such as ZO-1, occludin-1, and VE-cadherin [61,71]. This finally leads to tumor cell intravasation and systemic tumor cell dissemination [71].

The CXCL12/CXCR4 axis plays an important role in inducing the epithelial-to-mesenchymal transition (EMT) [61]. In this process, the overexpression of CXCL12 in tumor cells leads to their gaining the cancer stem cell-like (CSC-like) phenotype, which increases BC cell motility and starts their migration, eventually leading to invasion and metastasis [61,62].

The CXCL12/CXCR4 axis influences innate and adaptive immunity [72,73]. CXCL12 is of significant importance in the immune escape of breast cancer cells. It mobilizes immunosuppressive cells such as regulatory T cells and dendritic cells into a tumor’s microenvironment, thus limiting the efficiency of the immune response and enhancing cytotoxicity [62,74].

### 4.4. Contribution of CXCL12 to the Metastatic Spread of BC

As a result of all of the above-mentioned actions, only a small number of cancer cells invade the circulation. The presence of circulating tumor cells (CTCs) in the blood is supported by platelets, coagulation factors, signaling molecules, growth factors, and chemokines, which additionally determine the preference for metastatic target organs [75]. The CXCL12/CXCR4 axis supports the recruitment of circulating BCs into the brain, lungs, liver, and bones, as these organs are characterized by a high expression of CXCL12 [76,77,78,79]. The movement of cancer cells toward CXCL12 in metastatic sites is determined by the gain of CXCR4 expression and the loss of CXCL12 on the primary tumor at the same time, enabling movement of tumor cells, along with the CXCL12 gradient, toward the metastatic niche [66].

## 5. Coagulation

### 5.1. Tissue Factor (TF)—A Potent Activator of the Coagulation Cascade

The tissue factor is a transmembrane glycoprotein that binds factor VII (FVII) and forms the TF–FVIIa complex, which is responsible for initiating the coagulation cascade, eventually leading to the generation of thrombin, the activation of platelets, and the formation of insoluble clot [80,81]. The molecule comprises three domains: extracellular, transmembrane, and cytoplasmic. The procoagulant activity is attributed to the extracellular domain, while the cytoplasmic domain is involved in cell signaling [82]. TF is expressed on a broad range of cells, but it is mostly found in perivascular space, where it establishes vascular integrity [80]. Its expression can be induced by hepatocyte growth factor (HGF), tumor growth factor-β (TGF-β), VEGF-A, interferon-γ (IFN-γ), tumor necrosis factor-α (TNF-α), interleukin-6 (IL-6), IL-1β, IL-33, histamine, and CD40-ligand [80]. Furthermore, TF can be expressed in various cancers, including colon, pancreas, lung, head and neck, urothelium, and breast cancers [80,83].

### 5.2. The Role of Coagulation in BC

Patients with BC are characterized by higher concentrations of TF compared to healthy individuals. Furthermore, the blood levels of TF are associated with its expression on cancer cells, as well as on the stromal cells surrounding the tumor [83,84,85]. TF expression is much higher in metastatic cells than in the primary tumor [80]. The expression of TF is suspected to be a prognostic factor for BC patients; however, the results of related studies remain contradictory [83,84,85,86].

### 5.3. Interactions of TF with Tumor and Tumor Microenviroment

TF expression in cancer can be upregulated by mutations in the tumor suppressor gene TP53 and the proto-oncogene KRAS, which activate the mitogen-activated protein kinase (MAPK) and PI3/AKT signaling pathways, subsequently leading to the upregulation of TF on cancer cells [80]. Furthermore, TF expression is also stimulated by HIF-1α and VEGF-A [80]. Activation of intracellular signaling in TF-expressing cells contributes to cancer invasion and metastasis by initiation of the blood coagulation cascade or in a thrombin-independent way [84,87]. Moreover, TF, together with FVIIa, boosts tumor development by activating protease-activated receptor type 2 (PAR-2) or by enhancing the expression of VEGF-A [87]. Thrombin also stimulates tumor progression, angiogenesis, and metastasis by activating PAR-1 and by generating fibrin [88]. Not only do tumor cells express TF, but so do the surrounding stromal cells [88]. In tumor stroma, CAFs have the leading role in creating a tolerant environment for the spread of cancer, facilitating extravasation, extracellular remodeling, cell differentiation, and angiogenesis [88].

### 5.4. Link between Coagulation and Angiogenesis

The contribution of TF to angiogenesis is complex and involves different pathways and processes. It can upregulate the expression of the most potent proangiogenic molecule—VEGF—in CAFs, cancer, and ECs [87,89,90]. Furthermore, TF leads to an increase in the expression of angiogenesis triggers, such as IL-8 and fibroblast growth factor-4 (FGF-4), or downregulates angiogenesis inhibitors, such as thrombospondin-1 or thrombospondin-2. These actions contribute to the angiogenic switch [2,12] and may happen directly by TF–FVIIa signaling via PAR-2 or indirectly by a thrombin-dependent release of proangiogenic factors from activated platelets [12,13]. PAR-2 activation is responsible for tumor cell proliferation, migration, invasion, pro-survival activities, and, eventually, metastasis formation [2,15,16].

### 5.5. Contribution of TF to the Metastatic Spread of BC

EMT occurring in cancer progression, together with platelets, induces the overexpression of TF in BC cells, leading to the release of tumor cells into the bloodstream [91,92]. These cells play an important role in tumor dissemination, owing to their enhanced procoagulant properties [93]. TF expressed on CTCs induces a coagulation cascade in space around these cells that traps CTCs in platelet/fibrin mircothrombi, thus promoting their survival and spread to distinct organs [94].

The role of TF in metastasis formation involves distinct activities [83]. Both the coagulation-dependent and coagulation-independent functions of TF play roles in this process, although coagulation-dependent functions are considered to more selectively promote the early stages of metastasis [95,96]. These actions include adhesive interactions between cancer cells, platelets and fibrin, thrombin generation, and the protective effects of clots against natural killer cells [83]. Finally, TF induces the formation of metastases by enhancing fibrin deposition or by promoting the generation of coagulation proteases that activate PARs [87].

## 6. Metastatic Disease

### 6.1. Advanced Breast Cancer—The Formation of Metastases in Distant Organs

Early-stage BC generally has a good prognosis, with an overall survival of approximately 70–80%. However, the development of metastatic disease rapidly diminishes the prognosis, as metastasis accounts for 90% of deaths [61]. Bone is the most frequent site of BC metastasis formation, accounting for approximately 75% of all metastatic cases. Less than 25% of all patients in this group survive five years. The lungs constitute the second most frequent site for metastasis formation, followed by the liver. The five-year overall survival rate is 16.8 and 8.5%, respectively. Brain metastases account for approximately 15–30% of cases in metastatic disease [17]. Metastases in visceral organs are distinctive for hormone receptor-negative primary tumors, contrary to bone metastases, which are mostly the result of the metastatic spread of hormone-positive tumors [97]. The luminal A-like subtype generally has the lowest affinity to form metastases [97]. A study by Wu et al. that included a group of 243,896 BC patients revealed that patients with all subtypes were most prone to bone metastases, which were found in 8848 cases, followed by lung metastases in 4167 cases, 3434 liver metastases, and 1000 cases of brain metastases [98].

### 6.2. Concepts of Tumor Spread

There are two major models of tumor cell dissemination—the linear progression model and the parallel progression model. In the linear model [17], it is stated that metastasis formation occurs late during cancer development and is correlated with the tumor size [99]. In contrast, the parallel progression model assumes that metastatic spread, especially in BC, occurs early during tumor development, when various tumor clones detach from the primary tumor to settle parallelly in different secondary sites [17,99].

**Figure 2:**
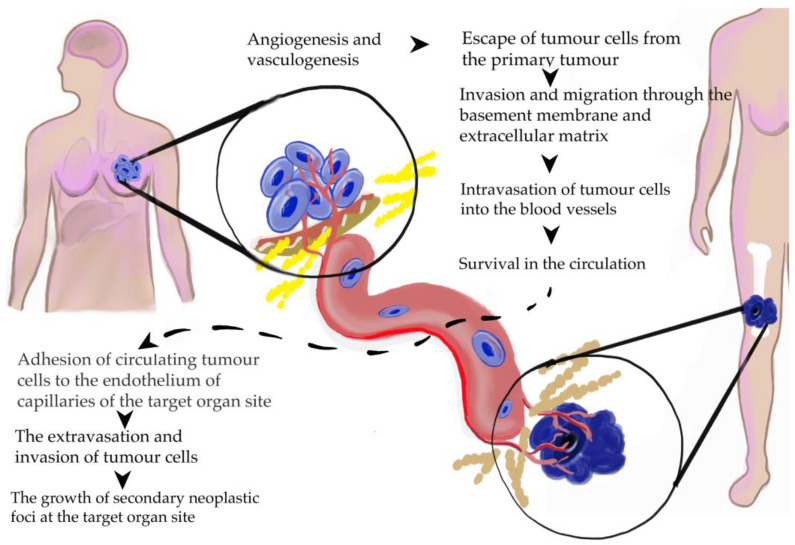
Main steps of metastasis formation in breast cancer [99].

Metastasis is a complex, multistep process, where a subset of cancer cells leave the primary tumor and colonize distant organs [18]. The most important actions occurring over metastatic spread are shown in Figure 2.

### 6.3. Systemic Dissemination of Cancer Cells

The TME facilitates cancer cell invasion and the survival and formation of metastases, consisting of cancer and stromal cells, the ECM, and blood vessels. Stromal cells encompass immune cells, CAFs, pericytes, and mesenchymal stem cells [18]. Among the immune cells in the TME, macrophages have the key role—they generate a proinflammatory microenvironment, thus promoting tumor growth. Furthermore, they trigger the formation of new blood vessels, the invasion of cancer cells, and the migration and restraint of anti-tumor immunity. Finally, they are involved in the extravasation, survival, and growth of tumor cells at the metastatic site. Another type of stromal cell, CAFs, have an important role in the TME—they promote angiogenesis, ECM remodeling, invasion, and, subsequently, metastasis [18]. There is relentless communication between tumor cells and the microenvironment, which occurs as a result of the secretion of growth factors, cytokines, and chemokines by the TME components. These actions bidirectionally modulate the behavior of cancer cells and the surrounding microenvironment, thus facilitating BC invasion, survival, and, eventually, metastases [18].

**Figure 3:**
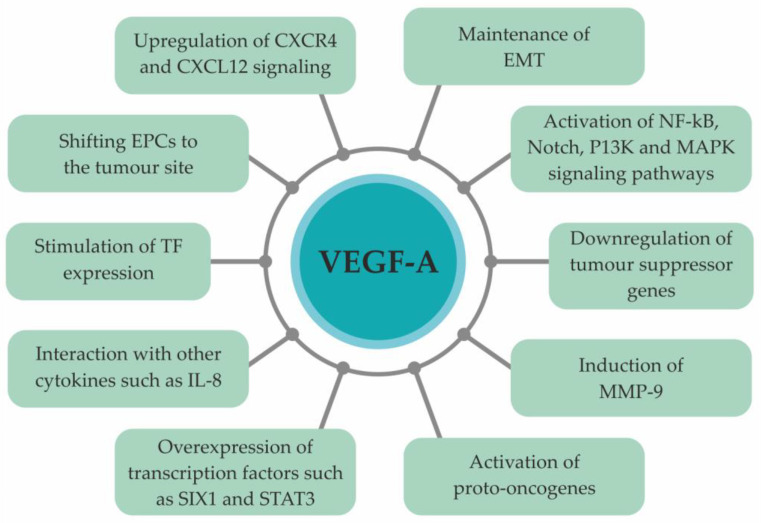
Role of VEGF-A in metastasis formation [100]. Abbreviations: VEGF-A, vascular endothelial growth factor A; EPCs, endothelial progenitor cells; TF, tissue factor; IL-8, interleukin-8; MMP-9, matrix metalloproteinase-9; EMT, epithelial-to-mesenchymal transition; CXCL12, C-X-C motif chemokine ligand 12; CXCR4, C-X-C receptor type 4.

EMT allows epithelial cells to obtain a mesenchymal cell phenotype [18], resulting in the loss of cell polarity and cell–cell adhesion and, simultaneously, the acquirement of a mesenchymal cell phenotype with great migrative, invasive, and anti-apoptotic potential [18]. During EMT, cancer cells undergo morphology alterations. Their epithelial markers, such as E-cadherin and cytokeratins 8 and 18, are downregulated, while mesenchymal markers on their surface are upregulated. These markers include N-cadherin, vimentin, fibronectin, and SMA [100]. EMT leads to increased BC cell motility and invasiveness, as well as to intensified angiogenesis and suppression of apoptosis in a VEGF-A- dependent manner [29,44]. The role of VEGF-A in BC metastasis formation is multifaceted and multidirectional (Figure 3).

Finally, implementation of treatment—chemotherapy, radiotherapy, targeted drugs or surgery—may induce exacerbation of metastatic processes through the process of ECM remodeling, activation of metastasis supporting factors, several immune cell types or ECs. Therapy might also lead to contribution of host and tumor cells to pre-metastatic niche formation [101].

### 6.4. Homing of Circulating Tumor Cells in Target Organs

Metastasis formation is a highly inefficient process—only a small number of tumor cells survive in the blood circulation and manage to reach the target organs [17]. Furthermore, the colonization of a second organ by disseminated tumor cells requires a complementary and adaptable microenvironment, which supports the homing of tumor cells to distant sites with characteristic attractant molecules [20]. There are two types of metastatic niches in target organs, namely, pre-existing and induced niches. In a pre-existing niche, the survival of cancer cells is enhanced by an organ-specific microenvironment [44]. On the contrary, a metastatic microenvironment can be created even during early stages by infiltration of different stromal cell types. The impact of cancer and stromal cells, together with cytokines, chemokines, growth factors, and other components of the ECM creates an organ-specific metastatic microenvironment that allows cancer cell settlement and the development of secondary tumors. These processes are complex and require strict involvement and cooperation of cancer cells and their microenvironment, which characterize by mutual interaction between proangiogenic, proinflammatory, procoagulatory, prochemattractive biomolecules [44].

### 6.5. Bones

The bone microenvironment supports the homing, survival, proliferation, and formation of metastases by CTCs [102]. As tumor cells reach bones, they affect the interactions between various cell types, including osteoblasts, osteoclasts, and hematopoietic stem cells, thus leading to alterations in bone homeostasis and, subsequently, the development of metastases [103]. Depending on histological and radiological aspects, bone metastases demonstrate osteolytic, osteosclerotic, or mixed types. Among them, osteolytic lesions are the most common; osteoclast activity is triggered, along with inhibition of osteoblasts’ actions, causing enhanced bone resorption and reduced bone formation [17]. These processes, supported by a “vicious cycle” of tumor-stroma interactions, are the molecular basis of overt bone metastasis formation [17,103].

Chemotaxis performs a remarkable function in terms of recruiting BC cells to bone [104]. CXCL12/CXCR4 signaling is one of the key players in this process [104]. CXCL12, secreted by bone marrow stromal cells (BMSCs) and osteoblasts, recruits CXCR4-expressing CTCs, directly stimulating their invasiveness and progression. Furthermore, CXCL12 is also responsible for homing circulating EPCs and reducing the expression of tight junction proteins [17,102,105], thus loosening the strength of connections between ECs [106]. As CTCs reach bones, crosstalk with resident stromal cells is crucial for their survival. Metastatic cells express integrin αµβ3, which facilitates their adhesion to ECM elements such as osteopontin, fibronectin, vitronectin, and thrombospondin. Moreover, the expression of integrin α4β1, which supports communication between tumor cells, stromal cells, and vascular cells by binding with intercellular adhesion molecule-1 (ICAM-1) and vascular cell adhesion molecule-1 (VCAM-1) [17].

Under physiological conditions, the receptor activator of nuclear factor–κB ligand (RANKL) and macrophage colony-stimulating factor (M-CSF) triggers osteoblasts to release collagenases and other bone matrix demineralizing proteases. Osteoblasts, in turn, differentiate into osteocytes, which contribute to the regulation of bone remodeling [105]. The invasion of BC cells to the bone disrupts the physiological process of bone remodeling. The normal processes of bone formation and mineralization by osteoblasts and bone resorption by osteoclasts become deregulated [1,104,105]. Metastatic cancer cells, after homing to the bone secrete RANKL, which is considered to be the leading regulator of bone homeostasis, stimulating osteoblasts to increase its release by producing cytokines and growth factors, such as parathyroid hormone-related protein (PTHrP), IL-6, VEGF-A, and TNF-α. This leads to osteolysis, causing a release of growth factors stored in the bone matrix, including TGF-β into the bone microenvironment [106]. These factors further stimulate osteoclasts to upregulate RANKL expression in osteoblasts, which subsequently promotes further bone destruction by osteoclasts [102,103]. As a result, osteolytic-type bone metastases are formed. On the contrary, when cancer cells release osteoblast-stimulating factors, osteosclerotic metastases emerge [102]. Activated osteoblasts release IL-6, monocyte chemoattractant protein-1 (MCP-1), VEGF-A, and macrophage inflammatory protein-2 (MIP-2), which trigger the development of metastatic cancer cells and communication with bone stromal cells [103].

During bone remodeling, matrix metalloproteases (MMPs) are released and degrade the ECM. MMP-2 and MMP-9 are mostly involved in this process. MMP-2 also activates TGF-β, which, in turn, supports the survival of BC cells in the bone microenvironment by inhibiting BC cell apoptosis [104]. Furthermore, MMPs regulate the release of VEGF-A, which supports angiogenesis at the metastatic site. VEGF type 1 receptors on osteoclasts, along with RANKL, also contribute to the dialogue between BC cells and osteoclasts, resulting in increased bone resorption [104].

After homing to the bone, most cancer cells remain in a state of dormancy, all the while not being adopted into the bone microenvironment [105]. Various mechanisms are assumed to maintain this state, encompassing the failure of proliferation in the bone microenvironment, insufficient vascularization, or inhibitory immune response. However, at the same time, dormant cancer cells acquire the capacity to initiate metastatic growth, utilizing the above-mentioned contributing mechanisms, and form macrometastases [17].

### 6.6. Lungs

Chemotactic molecules, cytokines, and proangiogenic factors are the main drivers of BC metastases to the lungs. Premetastatic niche formation facilitates tumor cells to colonize their target organ. Subsequently, the transendothelial migration of various cells and molecules, tumor microenvironment remodeling, and EMT, supported by angiogenesis, promote the survival and proliferation of cancer cells and, eventually, the formation of macrometastases [17].

The tumor microenvironment in the lungs is formed even before the arrival of cancer cells. The CXCL12/CXCR4 pathway has a principal role in premetastatic niche establishment. The CXCL12 chemokine is highly expressed in the lungs and attracts CXCR4-expressing cancer cells [107]. Various molecules and cells may affect its actions [108]. Some factors, such as TGF-β, may decrease the CXCL12 expression in the lungs and thus inhibit the development of metastases [107,109]. Moreover, CXCL12 supports the recruitment of TAMs into the lungs, which promotes secondary tumor growth and angiogenesis [61]. Furthermore, the amount of CXCR4 on a cell’s surfaces can be increased by VEGF-A, estrogens, hypoxia, CXCR2-expressing neutrophiles, and NF-κB [107].

The weak link of infiltration of BC cells into lungs is their migration through the vascular walls. CTCs, along with leukocytes and platelets, form emboli in lung capillaries. Contrary to bone marrow, pulmonary vessels are not fenestrated; moreover, these vessels are surrounded by a basement membrane and alveolar cells [108,110]. The tight cell–cell junctions between ECs impede the infiltration of lung parenchyma by CTCs. However, BC cells produce factors that enhance vascular permeability; these are SPARC, MMPs, COX2, and angiopoietin-like 4 protein (ANGPTL-4). Another essential for transendothelial migration molecules is nidogen (NID1), which initiates integrin signaling in ECs, thus disorganizing intercellular junctions to facilitate the migration of cancer cells through the vascular wall and their extravasation into the lung parenchyma [17]. Furthermore, VEGF-A from CTCs activates the Src-FAK pathway in ECs, leading to intensified expression of adhesion molecules and increased vascular permeability [110].

As BC cells reach the lungs, their expansion is promoted by cooperation with the lung microenvironment, immune cells, and bone marrow-derived dendritic cells (BMDCs), which is mediated by numerous cytokines, chemokines, and growth factors [110]. TAMs are one of the most important components of the TME. They promote tumor growth and invasion, as well as new vessel formation, by secreting numerous pro-angiogenic growth factors, which encompass VEGF-A, TNF-α, and TGF-β [61].

Hypoxia in primary tumor induces the expression of lysyl oxidase (LOX), which crosslinks collagen in the ECM in the lungs. This leads to the adhesion of CD11b+ myeloid cells and, subsequently, to the generation of MMP-2, which cleaves collagen, enabling recruitment of BMDCs and cancer cells to the TME in the lungs [108,109]. The secretory factors released to the TME by cancer cells recruit BMDCs into the lungs, which further facilitates the metastatic process [108].

Tenascin C (TNC), periostin (POSTN), and versican (VCAN) are ECM proteins whose actions are essential for BC cell seeding in lungs [107]. TNC increases the ability of BC cells to develop lung metastases by stimulating the Notch and Wnt signaling pathways [44]. POSTN expression in lung fibroblasts is triggered by TGF-β3, which binds to Wnt ligands [107] and promotes signaling among the cancer stem cells involved in the formation of metastases [44]. POSTN is also involved in escaping dormancy by BC cells [44]. VCAN is a protein secreted by BMDCs that promotes mesenchymal-to-epithelial transition (MET) in metastatic foci and, subsequently, the formation of macrometastases [44].

### 6.7. Brain

HER2+ or triple-negative BCs are more likely to form metastases in the brain than luminal cancers [111,112]. Young patients with high-grade, hormone receptor-negative tumors and with four or more lymph node metastases are at increased risk of developing brain metastases.

The CXCL12/CXCR4 axis is involved in the homing of BC cells in the brain, leading to the development of metastases [113]. CXCL12 is expressed by brain ECs, attracting CTCs that express CXCR4. Furthermore, CXCL12 increases vascular permeability. The binding of CXCL12 to CXCR4 activates the PI-3K/Akt signaling pathway, leading to BC cell migration through the blood–brain barrier (BBB) [113].

To pass through the BBB, CTCs must adhere to the vascular wall, establishing receptor–ligand interactions with ECs, and to extravasate into the brain parenchyma [112,113]. These interactions are mediated by selectins, expressed on ECs stimulated by inflammatory cytokines. Meanwhile, cancer cells express ligands for selectins [112,113]. The BBB is distinguished by the absence of fenestrations and the presence of tight junctions on ECs. This quality of the BBB, on the one hand, prevents cancer cells from entering the central nervous system, while on the other hand, also limits the immune response and the penetration of chemotherapeutic drugs, as metastatic cancer cells have already colonized the brain [113]. Endothelial tight junctions may be altered under particular circumstances, including metastasis formation [113]. The extravasation process takes place through paracellular transmigration (occurring through endothelial junctions, instead of through transcellular transmigration via single ECs). VEGF-A and CD 44 contribute to this process [113].

BC cells activate various cell types in the brain, among which, astrocytes and microglia are of exceptional importance. Activated astrocytes aggregate all over the metastatic foci, establishing direct contact with cancer cells and secreting a wide range of chemokines, cytokines, and interleukins [112]. Astrocytes support metastasis formation through the release of CXCL12. This chemokine decreases the expression of Kiss1 in BC cells, which subsequently leads to metastasis formation. High levels of CXCL12 in ECs increase vascular wall permeability and facilitate the migration of BC cells through the BBB [61]. Tumor cells alter the brain microenvironment through activation of the Akt/PI3K/mTOR, MAPK, and NF-κB signaling pathways [17]. This allows their growth and invasion, despite the neuroinflammatory reaction of microglia and astrocytes. The chemokines secreted by astrocytes, such as IL-6 and TGF-β, can also function as prooncogenic triggers for cancer cells, thus promoting the further migration and invasion of cancer cells [113]. Creating a favorable microenvironment is essential for metastasis development. One of the key factors in this process are MMPs, which not only create a premetastatic niche, but also activate growth factor signaling. MMP-9 and MMP-2, stimulated by overexpression of HER2 on cancer cells, degrade type IV collagen and, hence, the vascular basement membrane, enhancing the motility, invasion, and growth of metastatic cells [113].

Disseminated cancer cells stay in contact with brain microvascular ECs and grow along them, because in this way, they are provided with oxygen and the necessary nutrients. This process, occurring prior to the formation of a tumor’s own blood vessels, is termed vessel co-option and takes place irrespectively from sprouting angiogenesis [113]. The spread of BC cells along the vascular wall, as well as the interaction between BC cells, are mediated by the cell adhesion molecule LCAM. However, metastatic foci can increase their size only to a particular stage; their further growth requires angiogenesis activation [112].

As BC cells colonize the brain, they may remain in a state of dormancy, proliferation, or demise. Dormant cells can exist in the brain for many years until they are activated to start excessive growth and formation of macrometastases. Through EMT, cancer cells acquire the epithelial phenotype, which, along with interactions between tumor cells and ECM upregulation of particular signaling pathway, leads to the growth of macrometastases [113].

### 6.8. Liver

In around 50–70% of patients with metastatic BC, the liver is involved, but only in 5–12% it is develop as a primary metastatic site [114]. Unfortunately, in this group, the prognosis is poor, with the median survival being approximately two to three years [115]. TNBC and HER2+ BC are more likely to metastasize to liver [61].

Prior to CTCs reaching the metastatic site, the primary tumor secretes numerous molecules, which recruit bone marrow-derived hematopoietic progenitor cells (HPCs) to target organs and create a rich fibronectin niche before the homing of CTCs. Chemokine CXCL12, as in the previously described organs, is also expressed in the liver, which suggests that the CXCL12/CXCR4 axis plays important role in metastasis formation in the liver [61,116].

CTCs are held in the liver by attachment to the sinusoidal endothelial with the help of adhesion molecules such as cadherins, integrins, and CD44 [116]. Another important molecule involved in the adherence and transmigration of cancer cells via the endothelium is E-selectin, which is induced by TNF-α. This molecule seems to play a key role in the formation of BC liver metastases [61,116]. The fenestrated endothelium in hepatic sinusoids without a subendothelial basement membrane allows CTCs to migrate through the vessel wall in the liver [17,116].

When CTCs reach the target organ, a mesenchymal-to-epithelial transition occurs, resulting in the growth of metastases [116]. As MET takes place, cancer cells start to again express E-cadherin and N-cadherin. The latter involves the metastatic signaling pathway, together with fibroblast growth factor receptor and MMP-9. Furthermore, BC cells mediate HIF-1-regulated genes to survive and form macrometastases [116].

Initially, the early metastatic foci develop to a micrometastatic lesion. There are three different patterns of growth for metastases in the liver, specifically: replacement, desmoplastic, and pushing. The most frequent pattern is the replacement pattern, which is often recognized during the dormant or initial stage. In this pattern is hypoxia in the tumor; the TME is preserved, and the tumor is often incorporated into the sinusoidal vasculature, instead of forming its own blood vessels. The desmoplastic growth pattern is characterized by broad stromal remodeling at the tumor hepatocyte junctions [17], leading to the separation of normal hepatic tissue from invasive tumor cells. The pushing growth pattern is distinguished by the compression of hepatic cells by the metastatic tumor cells, without the formation of fibrin-rich stromal tissue [115].

## 7. Future Perspective

**Figure 4:**
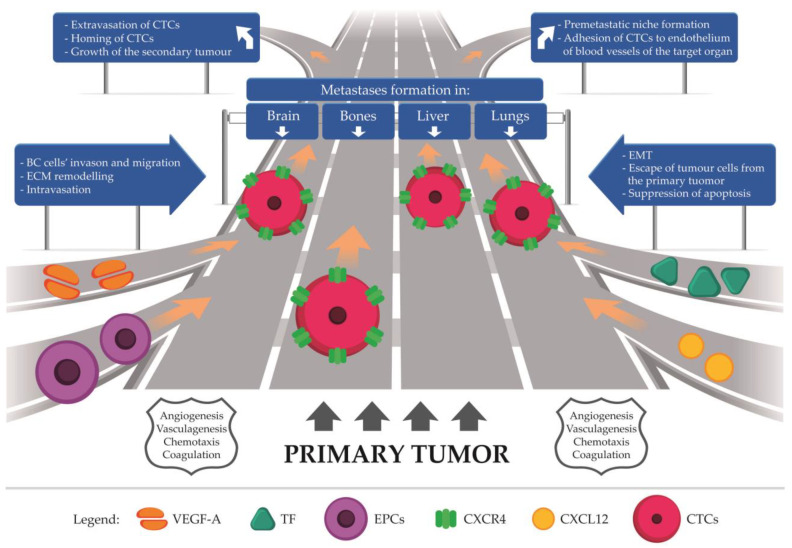
The authorial concept of metastasis formation in breast cancer. Circulating tumor cells (CTCs) released from the primary tumor form metastases in distant organs. This process is driven by angiogenesis, vasculogenesis, chemotaxis, and coagulation, which are mutually dependent and essential for metastatic spread. Abbreviations: VEGF-A, vascular endothelial growth factor A; TF, tissue factor; EPCs, endothelial progenitor cells; CXCR4, C-X-C motif chemokine receptor type 4; CXCL12, C-X-C motif chemokine ligand 12; CTCs, circulating tumor cells; BC, breast cancer; ECM, extracellular matrix; EMT, epithelial-to-mesenchymal transition.

As BC is a heterogenous disease, the behavior of particular molecular subtypes may vary during metastasis formation. The expression of hormone receptors on cancer cells or HER2+ tumors may affect this process, thereby facilitating or inhibiting metastatic spread. Furthermore, the development of metastatic lesions varies depending on the target organs, all of which create a particular microenvironment that enables tumor growth and invasion (Figure 4). Since metastasis formation is a complex process subject to numerous cellular interactions, and its exact mechanism has not been fully elucidated so far. To form metastatic foci, cancer cells must stay in the vascular system by adhering to the endothelium. Indisputably, creation of new blood vasculature is condicio sine qua non in cancer dissemination. High potential to angiogenesis is associated with decreased survival in women with breast cancer [25,86]. Therefore, a proper, complex understanding of new vessels network creation offers hope for providing effective treatments for breast cancer, thus increasing patient survival. However, scientists and clinicians should take into account serious side effects of antiangiogenic-targeted therapy including hypertension, proteinuria, bowel perforation, impaired wound healing, hemorrhage, and arterial prothrombotic state [117] and also the host, who is an independent element in this intricated puzzle.

## 8. Conclusions

Accurate recognition of the pathophysiological mechanisms of metastatic progression and their mutual relationships is crucial for understanding the explicit course of breast cancer systemic spread. Each of the described processes—angiogenesis, vasculogenesis, coagulation, and chemotaxis—is of crucial importance; furthermore, each propels the others, leading to the acceleration of metastasis formation. This should be taken into account by developing and establishing new therapies or modifications of already existing ones, which would subsequently improve BC patients’ overall survival.

## Figures and Tables

**Table 1:** The clinico-prognostic characteristics of the molecular subtypes of breast cancer [5,11].

	Luminal A-Like	Luminal B-Like	HER2- Overexpressing	Triple-Negative
**ER**	+	+	-	-
**PR**	≥20%	<20% ^1^	-	-
**HER2**	-	+ ^1^	+	-
**Ki-67**	<20%	≥20% ^1^	all	all
**Grade**	Low	Low/High	High	High
**Frequency**	30–40%	20–30%	12–20%	15–20%
**Local–Regional** **Recurrence**	0.8–8%	1.5–8.7%	1.7–9.4%	3–17%
**Prognosis**	Favorable	Intermediate	Unfavorable	Unfavorable/Poor

Abbreviations: ER, estrogen receptor; PR, progesterone receptor; HER2, human epidermal growth factor 2; Ki-67, proliferation index; ^1^ and/or.

**Table 2:** Involvement of various cytokines in angiogenesis [23,28,29,30,31,32,33,34,35,36].

Cytokines and Growth Factors Involved in Angiogenesis	Role/Action
**EGFR**	- upregulates of VEGF-A and other proangiogenic factors; - increases tumor cells proliferation and migration through EGFR-Ras/Raf/MEK/ERK and EGFR-PI3K/AKT pathways.
**bFGF**	- promotes tumor progression and metastases; - increases motility and invasiveness of BC cells; - stimulates proliferation, migration, and differentiation of endothelial cells; - increases production of proteases; - promotes of integrin and cadherin receptor expression; - supports communication across intercellular gap junctions.
**IL-8**	- stimulates the proliferation and survival of ECs; - upregulates MMPs in ECs; - supports recruitment of neutrophils into the tumor tissue; - promotes modification of expression of cells-adhesion molecules in BC cells and neutrophils.
**VEGF-A**	- the most potent proangiogenic molecule; - promotes proliferation and migration of ECs; - increases vascular permeability; - stimulates actin rearrangement; - influences gap junction; - enhances tumor cells extravasation;
**TNF- α**	- increases the expression of angiogenic factors, such as VEGF-A, bFGF, IL-8 in ECs; - proinflammatory cytokine; - induces cells survival, proliferation, and metastasis.
**MMPs**	- degrade and remodel ECM; - enhance ECs migration; - promote initiation of angiogenesis.

Abbreviations: bFGF, basic fibroblast growth factor; EGFR, epidermal growth factor receptor; VEGF-A, vascular endothelial growth factor A; IL-8, interleukin 8; MMPs, matrix metalloproteases; ECM, extracellular matrix; ECs, endothelial cells; BC, breast cancer; TNF-α, tumor necrosis factor-α.

## Data Availability

Not applicable.
